# Molecular Survey of Hepatitis C Virus in the Touristic City of Mar Del Plata, Argentina

**DOI:** 10.1371/journal.pone.0044757

**Published:** 2012-09-24

**Authors:** Andrés C. A. Culasso, Mercedes Elizalde, Rodolfo H. Campos, Luciana Barbini

**Affiliations:** Cátedra de Virología, Facultad de Farmacia y Bioquímica, Universidad de Buenos Aires, Buenos Aires, Argentina; Centers for Disease Control and Prevention, United States of America

## Abstract

The global epidemiology of Hepatitis C Virus (HCV) may be roughly described by two groups of genotypes: the worldwide distributed ones (subtypes 1a, 1b, 2a and 3a, among others) and the endemic ones (subtypes 4a, 5a, 6a, among others). Epidemiological and population dynamic studies of the worldwide distributed genotypes have shown that subtypes 1a and 3a are common among intravenous drug users (IDUs) and that they are also in expansion in some countries. The molecular survey of HCV provides some clues about the epidemiological status of the infections in a local scale and the phylogenetic and demographic reconstruction analyses complement this study by inferring whether the infections of certain subtypes are in a steady state or expanding. Here, a molecular survey of the HCV variants that circulate in the touristic city of Mar del Plata (Buenos Aires, Argentina) was performed in samples obtained from 42 patients. The subtypes detected were 1a (32 patients), 3a (8 patients) and 1b (2 patients). The demographic history of subtype 1a inferred using the sequence data showed an exponential growth in the 1990′s. The period of viral expansion was delayed compared with that observed for the same genotype in other countries where the transmission was associated with IDUs. Also, the phylogeographic analysis of HCV-1a showed a statistically significant association between the location of the samples and the phylogeny, which may be the result of the local transmission of HCV in the city. The molecular analysis helped in the description of the complex epidemiological context of a touristic city, and pointed out that some sanitary measures should be taken in order to reduce the transmission of HCV (and maybe of HIV) among IDUs.

## Introduction

Hepatitis C Virus (HCV) is an enveloped single-stranded positive RNA virus [1] that currently infects approximately 3% of the world's population [2]. In about 80% of infections, HCV causes a silent chronic hepatic illness that can lead to fibrosis, cirrhosis and hepatocellular carcinoma [3]. Taxonomically, HCV is classified into six major genotypes and many subtypes [4] by means of phylogenetic analysis of its genomic sequences. The epidemiology of HCV shows some geographic features for the different subtypes. For example, subtypes 1a, 1b, 2a and 3a have a worldwide distribution, whereas subtype 4a shows an endemic distribution in Africa [5]. The use of time-varying coalescent models [6] and population skylines [7] allows the reconstruction of the demographic history of each subtype, which is related to the main transmission route [8], and the prediction of the future incidence of HCV in several countries [9], [10]. Some genotypes, such as 1b and 2c, are in a demographic plateau and their transmissions are associated with medical practices [10], [11], while others, such as 1a and 3a, are in expansion, being the main route of transmission associated with intravenous drug use (IDU). Since human immunodeficiency virus (HIV) has the same hematological route of transmission, HCV is usually detected in HIV-infected patients, although the HCV subtypes in the co-infection vary among different locations [12], [13].

The molecular survey of HCV provides some clues about the epidemiological status of HCV infections in a local scale. However, the reconstruction of the population dynamics is a better tool to establish whether HCV infections are either in a steady state or expanding. The information retrieved by this analysis not only has immediate application in local public health policies, but may also help in the modeling of the epidemiological features of the different HCV genotypes.

In the present work, we performed a molecular survey of the HCV variants that circulate in Mar del Plata and, using sequence data, we inferred the demographic history of those strains in a hypothetical complex model of a touristic city.

## Results and Discussion

### HCV-1a, 1b and 3a circulate in Mar del Plata

**Figure 1:**
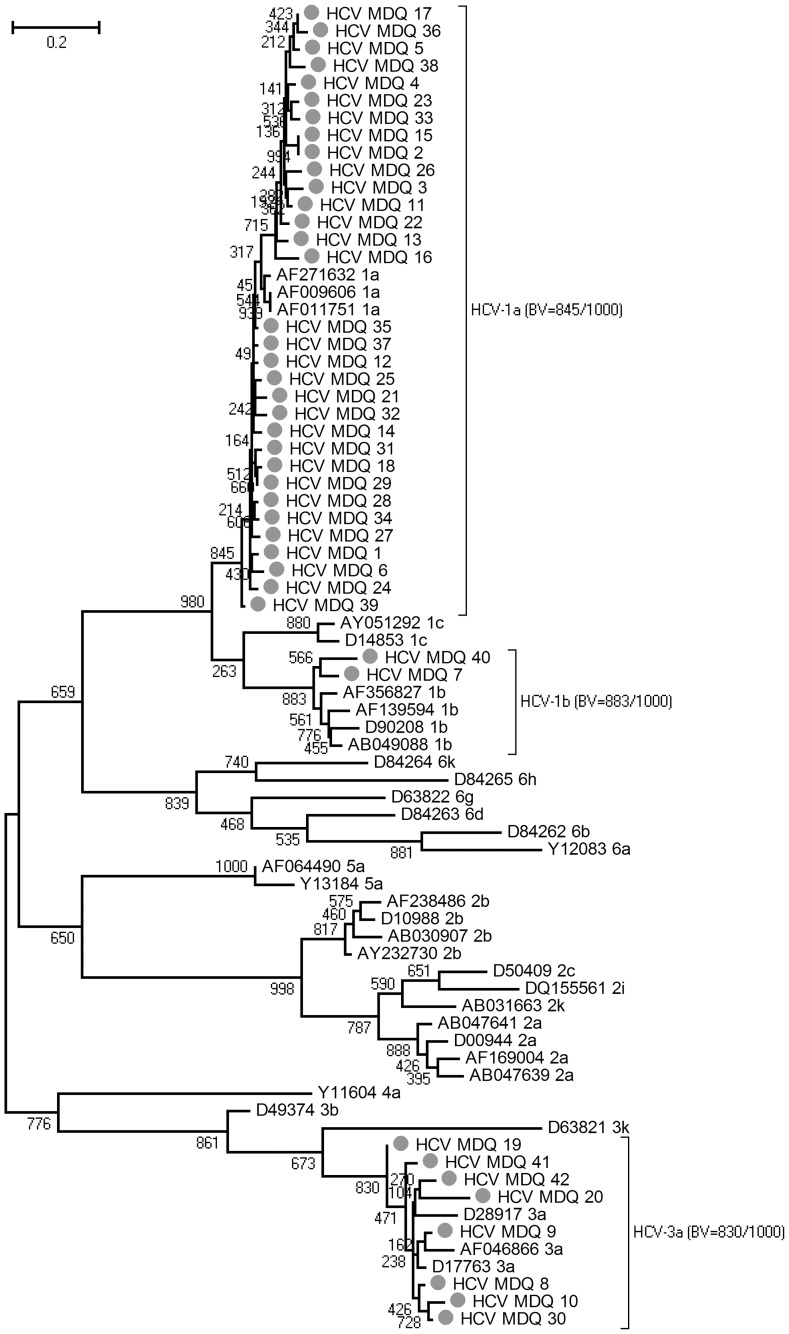
Maximum Likelihood Tree for the NS5B region obtained using GTR+Γ+I as a model of nucleotide substitutions. Gray circles : Samples from Mar del Plata (Buenos Aires, Argentina). The reference samples were named after their Genotype and Accession Number. The number above/below the branches represents the bootstrap value (over 1000 pseudo-replica). The relevant groups were highlighted with parentheses depicting the genotype and the bootstrap value (**BV**) of the genotype's clade.

In order to describe the HCV genotype distribution in Mar del Plata, 42 serum samples were studied by sequence analysis of NS5B and 5′NC regions. The phylogenetic analysis of these sequences with a dataset containing reference sequences produced a Maximum Likelihood tree in which 32 out of the 42 samples analyzed formed a highly supported group with the HCV-1a reference sequences, 2 grouped with the HCV-1b reference sequences and 8 grouped with HCV-3a (Figure 1). At least these three subtypes circulate in Mar del Plata, but the proportion of genotypes detected, which may reflect the genotype distribution, is different from that found in Argentina, where genotype 1b was the most prevalent [14]. Moreover, genotypes 1a and 3a were described associated with injected drug users (IDUs) [15]. However, since our analysis consisted of a molecular survey of HCV cases already detected, the proportions may be biased, and further randomized epidemiological studies on the general population must be carried out to describe the accurate HCV distribution and prevalence in Mar del Plata.

### HCV has arrived in Mar del Plata from multiple origins

**Figure 2:**
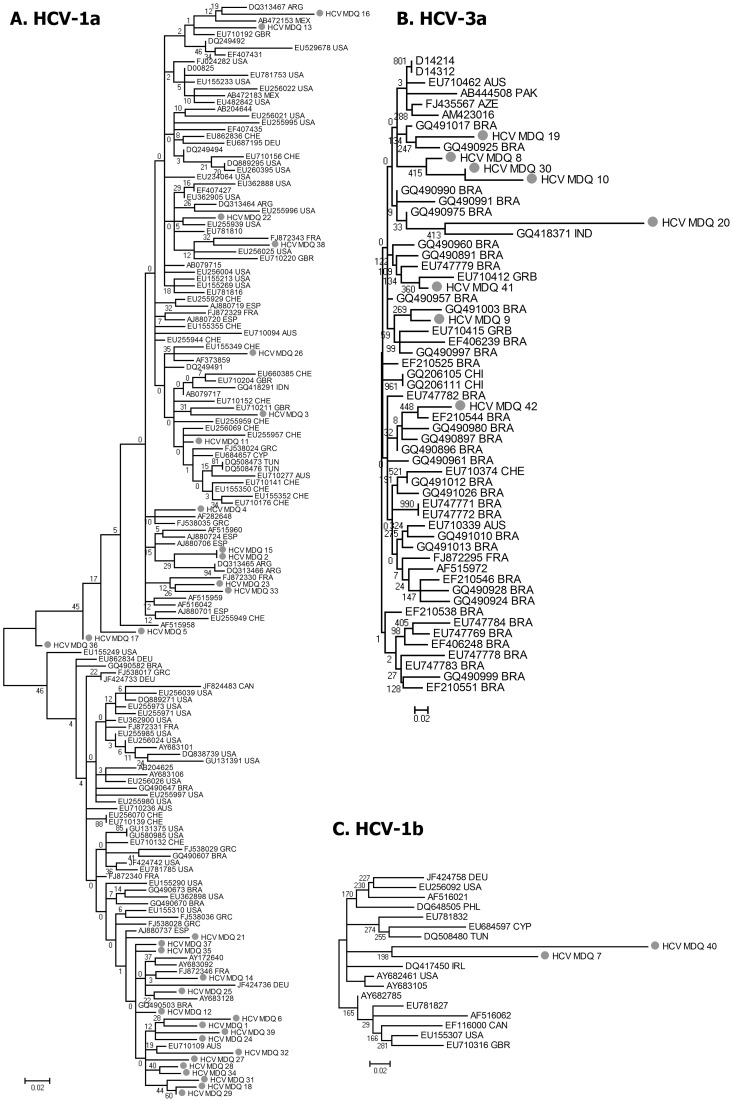
Maximum Likelihood Tree for the NS5B region in the context of the most similar sequences deposited at Genbank. A. Tree obtained for HCV-1a sequences (GTR+Γ+I). **B.** Tree obtained for HCV-3a (TrN+Γ+I). **C.** Tree obtained for HCV-1b (HKY+Γ+I). **Gray circles**: samples from Mar del Plata. The rest of the samples were the most similar sequences (obtained by BLAST of each sequence from Mar del Plata) named after their GenBank accession number, following the three-letter ISO code of the country: **ARG**: Argentina; **AUS**: Australia; **BRA**: Brazil; **CAN**: Canada; **CHE**: Switzerland; **CYP**: Cyprus; **DEU**: Germany; **ESP**: Spain; **FRA**: France; **GBR**: the United Kingdom; **GRC**: Greece; **IND**: Indonesia; **IRL**: Ireland; **MEX**: Mexico; **MTQ**: Martinique; **PHL**: the Philippines; **PRT**: Portugal; **TUN**: Tunisia; **USA**: the United States of America. The numbers either above or below the branches are the bootstrap value (over 100 pseudo-replica for HCV-1a, and 1000 pseudo-replica for HCV-3a and HCV-1b).

To assess the diversity of the three genotypes detected, we performed a “BLAST fishing” strategy where each sample was used as “bait” to retrieve (“fish”) the 10 most similar sequences at GenBank (Linux BASH scripts available upon request). The phylogenetic analysis of the sequences from Mar del Plata in the context of the retrieved sequences, showed that neither HCV-1a nor HCV-3a (from Mar del Plata) formed a monophyletic group (Figure 2 A and B). The two sequences obtained for HCV-1b formed an unsupported cluster (Figure 2 C), but since only two out of the 42 samples were genotype 1b, some degree of phylogenetic relationship between these samples is not surprising. These results suggest that the introduction of HCV in the community occurred from diverse sources even within the HCV-1a and HCV-3a subtypes.

### HCV-1a may have dispersed mainly in the 1990′s

**Figure 3:**
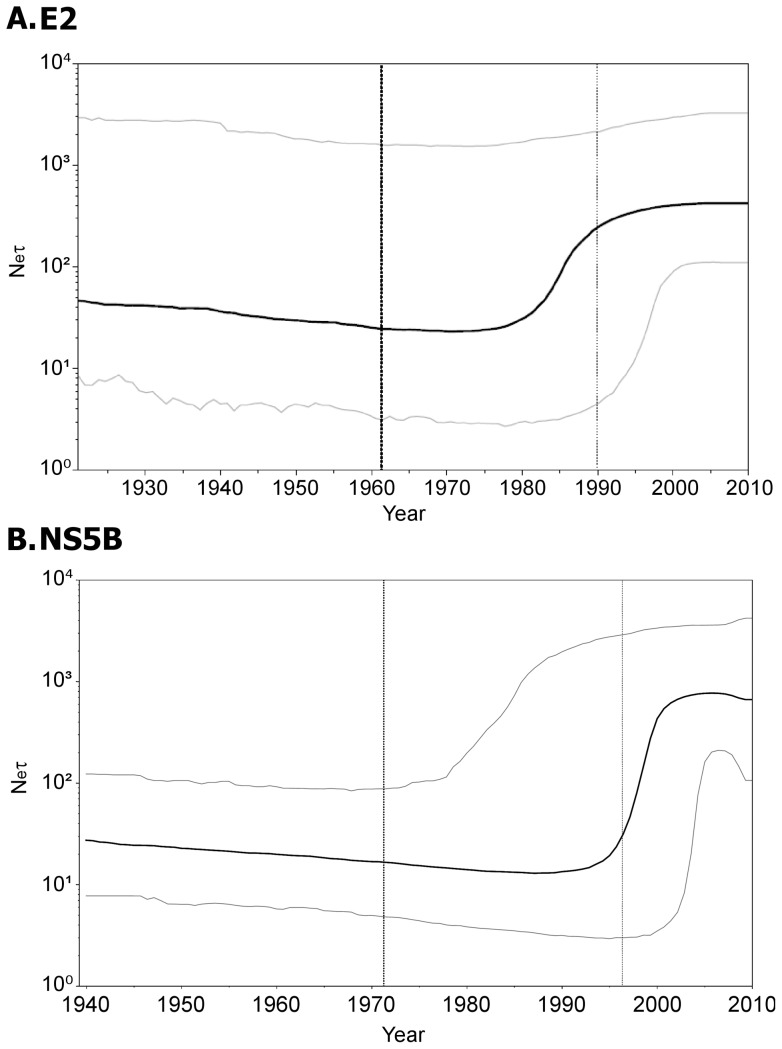
Bayesian Skyline Plots (BSPs) for the E2 and NS5B regions. The E2 BSP was obtained using 3.41×10^−3^ (SD = 4.53×10^−4^) s/s/y. The time of the most recent common ancestor obtained for the E2 analysis was set as a prior for the BSP of the NS5B region (lognormal distribution with a mean(log) = 3.85 and a SD(log) = 0.43). In both plots, the X axis represents the year B.C. and the Y axis represents the product between the Effective Population Number and the generation time (N_e_·τ) of HCV.

We further analyzed the demographic history of HCV-1a, using the Bayesian Skyline Plot (BSP). Under the priors set for the NS5B region (see *Demographic Reconstruction*), the time of the most recent common ancestor (t_MRCA_) for the HCV-1a samples was estimated to be 1953 (between 1920 and 1977) and the substitution rate was 1.42×10^−3^ substitutions per site per year (s/s/y; between 0.49 to 2.90×10^−3^ s/s/y). In addition, the BSP showed a period of exponential growth between ∼1985 and ∼1995 (Figure 3 B). For the E2 region, the t_MRCA_ of the samples was estimated to be 1966 (between 1921 and 1980) and the population dynamics also showed a period of exponential growth between ∼1970 and ∼1995 (Figure 3 A). The differences in the period of exponential growth between both analyses may be partly due to the number of sequences used in each dataset. The larger number of NS5B sequences (32) respect to that of the E2 sequences (11) provides a more lineage-rich sample. Moreover, in our experience, the nucleotide substitutions in the NS5B region resemble a drift process with a more clock-like behavior than the shift process often observed in the E2 region. Besides these differences, and the error interval of the analysis with the E2 dataset, which was wider than that obtained for NS5B, both results were compatible. The period of exponential growth obtained for the NS5B region was slightly delayed as compared to those previously described for HCV-1a both in developing countries [16] and in countries where its dispersion is thought to be related to the abuse of intravenous drugs [15].

We focused the research on HCV-1a because it is the main genotype detected, but similar analyses carried out in HCV-3a NS5B sequences showed results similar to those obtained for HCV-1a E2 (data not shown). We decided to make no further speculation about HCV-3a due to the low number of sequences and the short region analyzed for this subtype. Finally, subtype 1b was not analyzed since only two sequences were obtained for this subtype.

### The phylogeny of HCV-1a is associated with the geographic location

To statistically quantify the degree of diversification achieved by HCV-1a in Mar del Plata, a Bayesian Tip-association Significance test (BaTS) [17] was carried out. The phylogeny produced by the HCV-1a samples from Mar del Plata and the most closely related sequences from 19 countries (geographic “states”) from five continents (Oceania, Asia, Europe, Africa and America) (see *Phylogeography*) showed global evidence of geographic association assessed by the Association Index (AI) and the Parsimony Score (PS) (Table 1). In addition, the analysis of the Maximum Monophyletic Clade (MC) index, which is state specific, showed that the association was statistically supported (α = 0.01) for Tunisia, the United Kingdom, Venezuela and Mar del Plata. This result suggests at least some historical circulation of HCV-1a in these regions.

**Table 1:** Results of Bayesian Tip-association Significance testing (BaTS).

Statistic	observed mean	Lower 95%CI	Upper 95%CI	null mean	Lower 95%CI	Upper 95%CI	Significance
**AI**	9.0546	7.8203	10.2429	12.8462	12.1897	13.4408	**<0.001**
**PS**	71.7827	69.0000	75.0000	94.9082	92.0158	97.9100	**<0.001**
**MC(GBR)**	3.6785	3.0000	4.0000	1.3132	1.0475	1.9167	**0.01**
**MC(MDQ)**	4.3605	2.0000	8.0000	2.1644	1.8080	2.6858	**0.01**
**MC(TUN)**	2.0012	2.0000	2.0000	1.0145	1.0000	1.0785	**0.01**
**MC(VEN)**	7.9702	7.0000	8.0000	1.9393	1.4847	2.3998	**0.01**
**MC(DEU)**	2.0137	1.0000	3.0000	1.1382	1.0090	1.4942	**0.02**
**MC(IRN)**	1.9795	1.0000	3.0000	1.1463	1.0043	1.8212	**0.02**
**MC(ARG)**	1.0000	1.0000	1.0000	1.0085	1.0000	1.0517	1.00
**MC(BRA)**	1.0000	1.0000	1.0000	1.0131	1.0000	1.0753	1.00
**MC(CAN)**	1.1177	1.0000	2.0000	1.0198	1.0000	1.1097	1.00
**MC(CHL)**	1.0000	1.0000	1.0000	1.0000	1.0000	1.0000	1.00
**MC(COL)**	1.0000	1.0000	1.0000	1.0000	1.0000	1.0000	1.00
**MC(ESP)**	1.0383	1.0000	1.0000	1.0411	1.0000	1.2583	1.00
**MC(FRA)**	1.5293	1.0000	2.0000	1.0867	1.0007	1.3542	1.00
**MC(GRC)**	1.0005	1.0000	1.0000	1.0259	1.0000	1.1103	1.00
**MC(IDN)**	1.0000	1.0000	1.0000	1.0000	1.0000	1.0000	1.00
**MC(MEX)**	1.0005	1.0000	1.0000	1.0019	1.0000	1.0047	1.00
**MC(MTQ)**	1.0000	1.0000	1.0000	1.0000	1.0000	1.0000	1.00
**MC(PAK)**	1.0000	1.0000	1.0000	1.0000	1.0000	1.0000	1.00
**MC(PRT)**	1.3852	1.0000	2.0000	1.1493	1.0115	1.6073	1.00
**MC(USA)**	1.0000	1.0000	1.0000	1.0063	1.0000	1.0113	1.00

Association Index (**AI**) and Parsimony Score (**PS**) test the global association between a trait and tree topology, taking into account the level of uncertainty in the phylogenetic reconstruction. The Monophyletic Clade (**MC**) index tests which traits are associated with phylogeny. The observed mean and its associated 95% confidence intervals (**Upper and Lower CI**) were obtained by analyzing trees sampled during the Bayesian phylogenetic reconstruction. The null mean and its associated confidence intervals were obtained after randomly distributing the traits in the phylogeny (100 replica). The significance level is the **p** value for the statistical hypothesis test for equality between the index observed and that expected under no association. Each state was defined following the three-letter ISO code of the country where the sample was collected (see *Phylogeography*).

Since the samples from Mar del Plata came from different sources (institutions/physicians) and there is a measurable degree of geographic association, we had to rule out the hypothesis of sample-source phylogeny associations. Thus, we carried out another BaTS analysis using only the samples from Mar del Plata, and tested the correlation between the sample source and the phylogeny. This test showed no association between the sample source and the phylogeny (Table S1).

## Conclusion

The molecular analysis of the samples obtained from the epidemiological surveillance of HCV provided some clues about certain features of the epidemiology of HCV in the touristic city of Mar del Plata (Argentina). Most of the samples analyzed belonged to genotypes 1a and 3a, which are known to be associated with the current emergence of HCV usually by needle sharing by IDUs [15]. Furthermore, the HIV status of 21 out of 42 patients was known and almost all of them (20 out of 21) were positive for HIV test. The co-infection with HIV, another blood-borne pathogen, may also be explained by this route of transmission. The HCV-1a/-3a – HIV combination has also been reported in co-infected patients in other parts of Argentina [12], which suggests that such combination may be a hallmark of the IDU route of transmission in the region.

The sequence diversity observed for HCV suggests the multiple introductions of the viral variants that currently circulate in Mar del Plata. However, the level of association between the phylogeny of HCV-1a and the geographic localization of the samples may be the result of local transmission.

The demographic reconstruction analysis of HCV-1a, although not conclusive, showed a period of exponential expansion of the HCV population in the 1990′s, which is delayed compared with the estimated time of the global popularization of the use of intravenous drugs in both industrialized and developing countries [16].

The current official information about drug abuse of resident people in the region (available at http://www.observatorio.gov.ar/especificos/especificos-adicionales/La_situacion_epidemiologica_en_Argentina_2011.pdf) indicates that Mar del Plata city shows no special features compared with both Buenos Aires province and the rest of Argentina (the state-level and country-level statistics). This information also reports no increase in the use of illicit drugs (with the exception of marihuana) in the period between 2001 and 2009. Unfortunately, there is no reliable information about drug consumption in the 1990′s, which is the time pointed out by our analyses as the time of dissemination of HCV-1a infections. Finally, the phylogenetic, population dynamic and phylogeographic analyses suggest that tourism (around one million people along the summer vacations) may have facilitated the introduction of the IDU-associated HCV strains in the city, but that, once in the community, the virus was transmitted by the already existing dissemination routes. The population dynamics reconstructed for HCV-1a suggests that the dispersion of the virus in Mar del Plata occurred later than in industrialized countries.

Since the use of illicit drugs may be responsible for the dissemination process, sanitary measures should be taken in order to reduce the transmission of HCV (and maybe HIV) among IDUs.

## Materials and Methods

### Ethical Statement

Written informed consent to participate in this study was obtained from all patients. The study protocol was approved by the ethics committee of the “Facultad de Farmacia y Bioquímica de la Univerisdad de Buenos Aires” (record number 732575/2010) in accordance with the 1975 Helsinki Declaration.

### Patients and Samples

Serum samples from 42 HCV diagnosed patients were included in this survey. The study was designed as a retrospective molecular survey of HCV circulating in Mar del Plata, and thus the inclusion criteria were the geographical location (stable Mar del Plata population) and a previous detection of HCV by PCR. Samples were from four sources: 11 (sequences HCV_MDQ_1 to 11) from the *Hospital Regional Interzonal de Agudos Dr. Oscar Allende* of the city of Mar del Plata (Buenos Aires, Argentina) (one of which was negative for HIV test and 10 of which had an unknown status), 14 (sequences HCV_MDQ_12 to 25) from the National Institute of Epidemiology (Mar del Plata) (five of which were positive for HIV test and nine of which had an unknown status), 5 (sequences HCV_MDQ_26 to 30) provided by a Municipal physician (three of which were positive for HIV test and two of which had an unknown status) and finally 12 (sequences HCV_MDQ_31 to 42) from a local penitentiary institution (all of which were positive for HIV serologic test). The available information about the patients is listed in Table S2.

### PCR and Sequencing

The serum samples were subjected to RNA extraction with the TRIzol Reagent (Invitrogen, Catalog number: 15596-026) following the protocol provided by the manufacturer.

Then, the RNA was retrotranscribed into cDNA with Random Hexamer Primers (Biodynamics, Catalog number: B070-40) using the Moloney Murine Leukemia Virus Retrotranscriptase enzyme (M-MLV RT, Invitrogen, Catalog number: 28025-013).

The cDNA was used as template for three protocols of nested PCR targeted against the 5′ non-coding (5′NC), the NS5B, and the E2 regions, using the Recombinant Taq Polymerase enzyme (Invitrogen, Catalog number: 11615-010). The primers and protocol for the 5′NC and NS5B regions are described elsewhere [18], [19]. Briefly, the PCR conditions for the 5′NC region were set according to the enzyme manufacturer, using the buffer supplied with the enzyme, 1.25 mM of Mg^++^, 0.25 mM of each dNTP, and 0.625 pmol/µl of each primer. For both PCR rounds, the same thermocycler program was used: an initial step of denaturation at 94°C for 1 min, then 25 cycles consisting of one step of denaturation at 94°C (5 sec), a step of annealing at 50°C (35 sec) and a step of extension at 68°C (2.5 min) and, after the cycles, a final step of extension at 68°C (10 min). The first round was carried out with the forward 5NC-ES (5′ CTG TGA GGA ACT ACT GTC TT 3′) and reverse 5NC-EA (5′ ATA CTC GAG GTG CAC GGT CTA CGA GAC CT 3′) primers. Then, the first round product was used as template for a second round of PCR with the forward 5NC-IS (5′ TTC ACG CAG AAA GCG TCT AG 3′) and reverse 5NC-IA (5′ CAC TCT CGA GCA CCC TAT CAG GCA GT 3′) primers. These primers produce an amplicon of ∼251 base pairs (from nucleotides 63 to 313 in AF009606).

The PCR conditions for the NS5B were set according to the enzyme manufacturer, using the buffer supplied with the enzyme, 1.5 mM of Mg^++^, 0.2 mM of each dNTP, and 0.2 pmol/µl of each primer. For both PCR rounds, the same thermocycler program was used: an initial step of denaturation at 94°C for 1 min, then 40 cycles consisting of one step of denaturation at 94°C (20 sec), a step of annealing at 55°C (30 sec) and a step of extension at 72°C (1 min) and, after the cycles, a final step of extension at 72°C (10 min). The first round was carried out with the forward NS5B-1 (5′ TGG GGT TCT CGT ATG ATA CCC 3′) and reverse NS5B-2 (5′ CCT GGT CAT AGC CTC CGT GAA 3′) primers. Then, the first round product was used as template for a second round of PCR with the forward NS5B-3 (5′ GAT ACC CGC TGC TTT GAC TC 3′) and reverse NS5B-4 (5′ CCT CCG TGA AGG CTC TCA 3′) primers. These primers produce an amplicon of 367 nucleotides (from nucleotides 8625 to 8259 in AF009606).

The PCR conditions for the E2 region were set as for the NS5B region. For both PCR rounds, almost the same thermocycler program was used: an initial step of denaturation at 94°C for 5 min, then 30 or 40 cycles (round one or two respectively) consisting of one step of denaturation at 94°C (1 min), a step of annealing at 55°C (1 min) and a step of extension at 72°C (1 min) and, after the cycles, a final step of extension at 72°C (10 min). The first round of PCR was carried out with the forward E2-ES (5′ GGA TAT GAT GAT GAA CTG GTC 3′) and reverse E2-EA (5′ ATG TAC AGC CGA ACC AGT TG 3′) primers. The first round product was used as template for a second round of PCR with the forward E2-IS (5′ GGA CAT GAT CGC TGG TGC TCA 3′) and reverse E2-IA (5′ TCC GCA CAC TTT GGT GAA TCC 3′) primers. These primers produce an amplicon of 661 nucleotides (from nucleotides 1376 to 2036 in AF009606, which include the amino terminal half of E2 region).

All PCR products were run on 1% agarose gel, stained with ethidium bromide, and evaluated under UV light. The amplified DNAs were purified from the PCR reaction and then sequenced in both senses, using the internal (second round) PCR primers by Macrogen, Inc. (Seoul, Korea).

### Sequences

The sequences analyzed in this work are available at GenBank under the accession numbers JQ272915 to JQ272931 (for the E2 region which conforms the E2 dataset), JQ272932 to JQ272974 (for the NS5B region which conforms NS5B dataset) and JQ272975 to JQ273013 (for the 5′NC region).

### Genotyping

The samples were genotyped by sequence similarity analysis of the 5′NC region, and the subtypification was carried out by phylogenetic analysis of the NS5B sequences from the samples. In both cases, the reference sequences were obtained from the NCBI Viral Genotyping Tool dataset (http://www.ncbi.nlm.nih.gov/projects/genotyping/view.cgi?db=3).

### Phylogeny

The sequences obtained were edited and prepared with BioEdit v 7.0.9 [20] and aligned using ClustalX 2.12 [21] along with the sequences downloaded from GenBank. For genotyping, the sequences downloaded belonged to complete reference genomes (see *Genotyping*). To test the monophyly of the samples from Mar del Plata, another dataset was prepared using the 10 non-repeating best hits in the BLASTN program (“nr” database) of each sequence from Mar del Plata. For each dataset, the best fit model of nucleotide substitution was selected using jModelTest v 0.1.1 [22], assessed by the Akaike Information Criterion. The phylogenetic relationships in each dataset analyzed were evaluated by the Maximum Likelihood methodology with PhyML 3.0 for Linux [23], setting the parameters obtained by the model selection program. The branch support was evaluated by non-parametric bootstrapping with 1000 pseudo-replica. The trees were prepared for publication using the TreeExplorer module of the program MEGA 4 [24].

### Demographic Reconstruction

The history of infections with HCV-1a was reconstructed using Bayesian Skyline Plots (BSPs) [7] implemented in the BEAST 1.7.1 software [25].

Since the analysis requires the viral sequences and a nucleotide substitution rate, our first approach was to use the sampling date of the E2 dataset to estimate the rate and the population dynamics simultaneously. After comparing the results of the analyses carried out with the actual sampling date and those carried out with shuffled dates, we concluded that the dataset was not time-structured and that the co-estimation of the rate was not possible (data not shown). Then, we downloaded an external dataset composed of 76 complete genomes from the USA, a country with many sequences with a known sampling date (Table S3 A), and produced a dataset covering exactly the same region as our E2 dataset. Using this dataset, it was possible to estimate the substitution rate for the E2 region as a normal distribution with a mean of 3.41×10^−3^ s/s/y and a standard deviation (SD) of 4.53×10^−4^ (Table S3 B). Using this prior (the normal distribution) as substitution rate, we estimated the time of the most recent common ancestor of the E2 sequences as a lognormal distribution with a mean of 3.85 and a SD f 0.43 (both in “log space”; see *Prior parameter Estimation* subsection for details).

The BSPs were run under the two molecular clock models: Strict and Relaxed Uncorrelated Lognormal. The runs were compared by Bayes Factors [26] and only the results obtained for the clock model that better represented the molecular data are presented.

Similar analyses were carried out for HCV-3a, using the HCV-1a substitution rate for NS5B, since the attempt to estimate the HCV-3a NS5B substitution rate was not possible due to the short period covered by the dated sequences deposited at GenBank for this genotype (data not shown).

All BEAST run logs were analyzed with TRACER version 1.5 (available from http://beast.bio.ed.ac.uk/Tracer) after discarding the initial 2% of the run length as burn-in. The achievement of the convergence was evaluated by visual inspection of the lack of trends in the trace of each parameter and by checking that the effective sample size (ESS) was 200 or higher for all the parameters.

### Prior parameter estimation

To estimate the distribution of the prior used in the actual analyses, calibration analyses were run using an external dataset. The density distribution of the model parameters (“meanRate” or “treeModel.rootHeight”) in the calibration BEAST runs were loaded in R (2.13.1 for Linux, available at http://www.r-project.org/). Then, the parameters of the normal, log-normal, gamma and Laplace distribution were estimated by Maximum Likelihood (MLE). These distributions were plotted over the density distribution of the actual parameter to graphically select the one which better represented the calibration run. Finally, the distribution selected and the parameters estimated were loaded in the prior section using the BEAUti tool provided by the BEAST software.

### Phylogeography

The association between phylogeny and geography was statistically assessed in a Bayesian framework implemented in the BaTS program [17], which takes into account the phylogenetic uncertainty in the analysis. A new dataset containing all the HCV-1a sequences from Mar del Plata and the 10 most closely related HCV-1a sequences (for each sample from Mar del Plata) with known sampling country were analyzed with MrBayes 3.1.2 [27]. Twenty states were defined, named after the geographic origin of the sample: ARG (Argentina, 3), BRA (Brazil, 3), CAN (Canada 4), CHL (Chile, 1), COL (Colombia, 1), DEU (Germany, 9), ESP (Spain, 4), FRA (France, 7), GBR (the United Kingdom, 13), GRC (Greece, 5), IDN (Indonesia, 1), IRN (Iran, 9), MDQ (Mar del Plata, 32), MEX (Mexico, 2), MTQ (Martinique, 1), PAK (Pakistan, 1), PRT (Portugal, 9), TUN (Tunisia, 3), USA (the United States of America, 2), VEN (Venezuela, 28). The model of nucleotide substitution was set to the General Time Reversible (GTR) Model, with heterogeneity in the substitution rate modeled with both a proportion of invariant sites and a gamma distribution of the substitutions (Γ+I), according with the analysis of jModelTest. The trees obtained were analyzed with BaTS to calculate the Parsimony Score (PS), the Association Index (AI) (for overall association) and the Monophyletic Clade (MC) (for each state) statistics. The expected value of the indexes under the non-association hypothesis was estimated by 100 randomized sets.

## Supporting Information

Table S1 Results of the BaTS analysis using the sample source as trait. Association Index (**AI**) and Parsimony Score (**PS**) test the global association between a trait and tree topology, taking into account the level of uncertainty in the phylogenetic reconstruction. The Monophyletic Clade (**MC**) index tests which traits are associated with phylogeny. The observed mean and its associated 95% confidence intervals (**Upper and Lower CI**) were obtained by analyzing trees sampled during the Bayesian phylogenetic reconstruction. The null mean and its associated confidence intervals were obtained after randomly distributing the traits in the phylogeny (100 replica). The significance level is the **p** value for the statistical hypothesis test for equality between the index observed and that expected under no association. Each state was defined by the source of the sample: state 0, patients from the penitentiary institution; state 1, patients from the “Instituto Nacional de Empidemiología”; state 2, patients from Municipal physicians; state 3, patients from the “Hospital Interzonal General de Agudos Dr. Oscar Allende”.
(XLS)

Table S2 List of available information for the patients analyzed. Sample Code: name of the sample as is shown in the trees. Sample Date: year (B.C.) of the sampling. Gender: M for male, F for female or N/A for not informed/available. Age: Age of the patient at the sampling time. HIV serology: serologic status of the patient for HIV diagnosis (N/A not available). Source: Institution that remitted the sample. 5′ UTR: Results of the genotypification by sequence similarity (NCBI Genotyping Tool) with the 5′UTR region. NS5B: Results of the subtypification carried out by phylogenetic analysis of NS5B region (See *Genotyping* section). “E2 sequenced?”: indicates if there are sequences available for the E2 region.
(XLS)

Table S3 A “Sequences”: accession number, name of the sample and sampling year of the external “calibration” dataset. B “BEAST Results”: results of the estimation of the substitution rate with the GenBank sequences. A. Sequences: List of the Genbank's accession number of the sequences used in the external calibration dataset. The Isolation Name and Collection Date of each Sample were obtained from the “Features” field of the Genbank data. B. BEAST Results: Summary of the results of the Bayes Factor Analysis carried out to test the models of strict and relaxed molecular clocks and the summary statistics of the estimation of the substitution rate parameters obtained with each clock model.
(XLS)
